# The complete chloroplast genome of *Ilex* ‘Tall Boy’, *Ilex aquifolium* × *Ilex latifolia* (Aquifoliaceae)

**DOI:** 10.1080/23802359.2020.1852898

**Published:** 2021-01-19

**Authors:** Fan Zhang, Yanwei Zhou, Hong Chen, Naiwei Li, Chuanyong Wang, Xiaoqing Lu, Yunlong Li

**Affiliations:** Jiangsu Key Laboratory for the Research and Utilization of Plant Resources, Institute of Botany, Jiangsu Province and Chinese Academy of Sciences, Nanjing, Jiangsu, China

**Keywords:** *Ilex* ‘Tall Boy’, complete chloroplast genome, phylogenetic analysis

## Abstract

Thecomplete chloroplast (cp) genome of *Ilex* ‘Tall Boy’, an important economic plant with ornamental and ecological values, was sequenced to investigate its phylogenetic relationship. The entire cp genome of ‘Tall Boy’ was 157,527 bp in length with 37.65% overall GC content, including a large single-copy (LSC) region of 87,044 bp and a small single-copy (SSC) region of 18,429 bp, which were separated by a pair of inverted repeats (IRs) of 52,054 bp. The cp genome contained 135 genes, including 90 protein-coding genes, 37 tRNA genes, and 8 rRNA genes. Phylogenetic analysis based on whole cp genome sequences showed that ‘Tall Boy’ is closest to *I. latifolia* Thunb. species.

*Ilex* ‘Tall Boy’ is an artificial hybrid between *I. aquifolium* L. and *I. latifolia* Thunb., which has been widely spread in southeastern China for its ornamental, ecological and economical values. However, due to the similar leave with other species and cultivars, it is difficult to be distinguished and identified by morphology (Yao et al. 2016). As an effective DNA molecular marker, the chloroplast genome has been widely used in genetic and evolutionary relationships studies in plants (Freitas et al. 2018). In present study, we determined the complete sequence of the chloroplast genome of *I.* ‘Tall Boy’ with bioinformatics analysis, which would be helpful for further research on the identification and classification of genus *Ilex* L.

Total genomic DNA of ‘Tall Boy’ was collected from Nanjing Botanical Garden, Mem. Sun Yat-sen (118°49′55″E, 32°3′32″N), Nanjing, China. The voucher specimen (NO. NBGJIB-Ilex-0007) was deposited in the Institute of Botany, Jiangsu Province and Chinese Academy of Science. Total DNA was extracted using the GMS16011.2.1 Kit (Genmed Scientifics Inc., USA). A paired-end library with an insert-size of 350-bp was constructed and sequenced on the Illumina NovaSeq system (Illumina, San Diego, CA, USA). In total, 4757.8 Mb of raw data (4499.8 Mb clean data) were obtained. De novo genome assembly and annotation were conducted by NOVOPlasty (Dierckxsens et al. 2017) and GeSeq (Tillich et al. 2017), respectively. The annotated cp genome was deposited in Genome Warehouse database (accession number: GWHAOTK01000000).

The whole cp genome sequence of ‘Tall Boy’ was 157,527 bp in length, including a large single-copy (LSC) region region of 87,044 bp and a small single-copy (SSC) region of 18,429 bp separated by two inverted repeats (IRs, including IRa and IRb) of 52,054 bp. The ‘Tall Boy’ cp genome contained 135 genes, including 37 transfer RNA genes, 8 ribosomal RNA genes and 90 protein-coding genes. Eight protein coding genes, four rRNA genes and seven tRNA genes are duplicated in the IR regions. A total of 12 genes contained one (10 genes) or two (*ycf3*, *clpP*) introns. The overall GC content of the cp genome was 37.65%.

To determine the phylogenetic status of ‘Tall Boy’, 11 other cp genome sequences were obtained from the Genebank database (Yao et al. 2016; Cascales et al. 2017; Park et al. 2019). A phylogenetic tree was constructed based on the complete cp genome of ‘Tall Boy’ and other reference genomes. The neighbor joining (bootstrap repeat is 10,000) and maximum likelihood (bootstrap repeat is 1000) were used for constructing phylogenetic trees using PhyML v3.0 (http://www.atgc-montpellier.fr/phyml/) (Liu et al. 2019). As excepted, the phylogenetic tree showed that ‘Tall Boy’ clustered into the *Ilex* section, and has more closely related to its male parent *I. latifolia* species (Figure 1). The cp genome sequence of ‘Tall Boy’ in this study will be useful for further analysis on molecular markers and molecular breeding.

**Figure 1.**
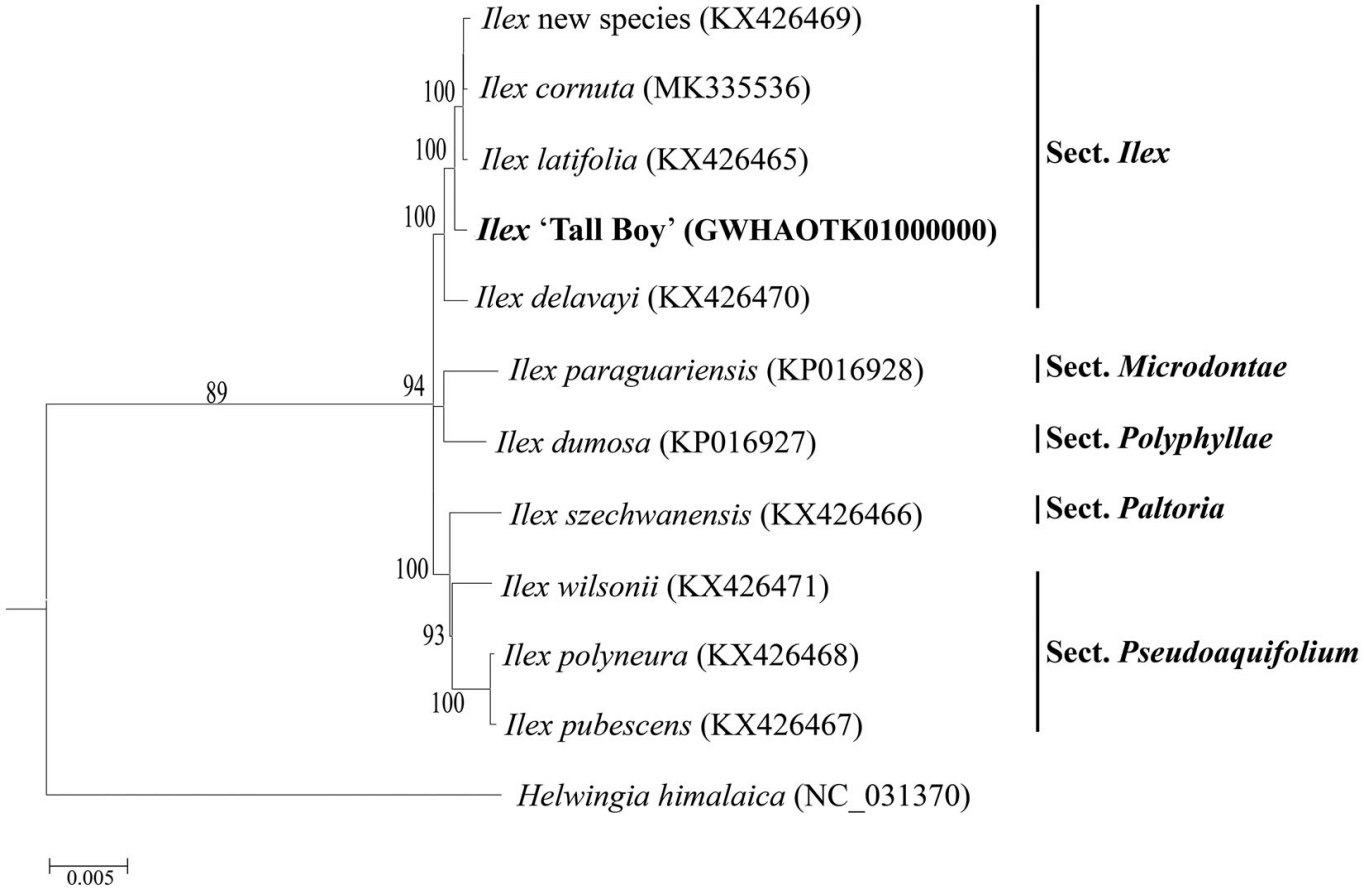
Maximum likelihood phylogenetic tree based on the sequences of Ilex ‘Tall Boy’ and other 11 complete chloroplast genomes. Section names were displayed in the right side of phylogenetic tree (Gottlieb et al. 2005; Jiang et al. 2017). Numbers on the nodes indicate bootstrap values.

## Data Availability

The complete chloroplast genome sequence of *Ilex* ‘Tall Boy’ is deposited in the Genome Warehouse (https://bigd.big.ac.cn/gwh) database under the accession number GWHAOTK01000000. The raw sequencing data is deposited in the Genome Sequence Archive database (https://bigd.big.ac.cn/gsa) under the accession number CRA003330.

## References

[CIT0001] Cascales J, Bracco M, Garberoglio MJ, Poggio L, Gottlieb AM. 2017. Integral phylogenomic approach over Ilex L. species from southern South America. Life. 7(4):47. 10.3390/life7040047 PMC5745560 29165335

[CIT0002] Dierckxsens N, Mardulyn P, Smits G. 2017. NOVOPlasty: de novo assembly of organelle genomes from whole genome data. Nucleic Acids Res. 45(4):e18. 28204566 10.1093/nar/gkw955 PMC5389512

[CIT0003] Freitas A, da Anunciação R, D’Oliveira-Matielo C, Stefenon V. 2018. Chloroplast DNA: a promising source of information for plant phylogeny and traceability. J Mol Biol Methods. 1:2.

[CIT0004] Gottlieb AM, Giberti GC, Poggio L. 2005. Molecular analyses of the genus Ilex (Aquifoliaceae) in southern South America, evidence from AFLP and ITS sequence data. Am J Bot. 92(2):352–369. 21652411 10.3732/ajb.92.2.352

[CIT0005] Jiang L, Xu K, Fan Q, Peng H. 2017. A new species of Ilex (Aquifoliaceae) from Jiangxi Province, China, based on morphological and molecular data. Phytotaxa. 298(2):147–157.

[CIT0006] Liu J, Champer J, Langmüller AM, Liu C, Chung J, Reeves R, Luthra A, Lee YL, Vaughn AH, Clark AG, et al. 2019. Maximum likelihood estimation of fitness components in experimental evolution. Genetics. 211(3):1005–1017. 30679262 10.1534/genetics.118.301893 PMC6404243

[CIT0007] Park J, Kim Y, Nam S, Kwon W, Xi H. 2019. The complete chloroplast genome of horned holly, Ilex cornuta Lindl. & Paxton (Aquifoliaceae). Mitochondr DNA Part B. 4(1):1275–276.

[CIT0008] Tillich M, Lehwark P, Pellizzer T, Ulbricht-Jones ES, Fischer A, Bock R, Greiner S. 2017. GeSeq–versatile and accurate annotation of organelle genomes. Nucleic Acids Res . 45(W1):W6–W11. 28486635 10.1093/nar/gkx391 PMC5570176

[CIT0009] Yao X, Tan Y-H, Liu Y-Y, Song Y, Yang J-B, Corlett RT. 2016. Chloroplast genome structure in Ilex (Aquifoliaceae). Sci Rep. 6(1):28559. 27378489 10.1038/srep28559 PMC4932625

